# Quality of life can both influence and be an outcome of general health perceptions after heart surgery

**DOI:** 10.1186/1477-7525-5-27

**Published:** 2007-05-24

**Authors:** Lars Mathisen, Marit H Andersen, Marijke Veenstra, Astrid K Wahl, Berit R Hanestad, Erik Fosse

**Affiliations:** 1 The Interventional Centre, Faculty Division Rikshospitalet, Faculty of Medicine, University of Oslo, N-0027 Oslo, Norway; 2 Dept of Thoracic and Cardiovascular Surgery, Rikshospitalet-Radiumhospitalet Medical Center, Sognsvannsveien 20, N-0027 Oslo, Norway; 3 Dept of Surgery, Rikshospitalet-Radiumhospitalet Medical Center, Sognsvannsveien 20, N-0027 Oslo, Norway; 4 Dept. of Biostatistics, Rikshospitalet-Radiumhospitalet Medical Center, Sognsvannsveien 20, N-0027 Oslo, Norway; 5 The Institute of Public Health/Faculty of Social Sciences, University of Bergen, N-5020 Bergen, Norway

## Abstract

**Background:**

Our aim was to investigate the existence of a reciprocal relationship between patients' assessment of quality of life and their appraisal of health. If present, this relationship will interfere with the interpretation of heart surgery's effect on overall quality of life.

**Methods:**

Path analysis was used to investigate reciprocal causal relationships between general health perceptions and overall quality of life before and after heart surgery. Longitudinal data from a study of coronary artery bypass surgery were used to model lagged, cross-lagged, and simultaneous paths over four time-points of assessment from before surgery to one year afterwards. The conceptual framework for the analysis was the Wilson and Cleary causal pathway model. General health perceptions were measured with the Short Form 36. Overall quality of life was measured with i) a single question regarding life satisfaction and ii) the multi-item Quality of Life Survey.

**Results:**

Acceptable model fit was obtained for reciprocal causation between general health perceptions and overall quality of life. Regression coefficients changed over different phases of rehabilitation. Serial correlation accounted for much of the variance within variables over time.

**Conclusion:**

The present analysis demonstrates that unidirectional models of causality are inadequate to explain the effect of heart surgery on overall quality of life. Overall quality of life can causally influence as well as be an outcome of health status after coronary artery bypass surgery.

## Background

Practice guidelines for chronic stable angina and for the coronary artery bypass operation target improvement of survival and symptomatic relief of angina [1], with improvement of the quality of life (QoL) as an expected secondary outcome [2]. Although a consensus exists on the subjective nature of QoL, the issues of what to measure and how to interpret the results remain areas of controversy [3]. Meanwhile, the number of papers returned from a database query of 'QoL and coronary artery bypass surgery' average one per week for every year of the last five years, and clinicians face the task of judging the validity and significance of this research [4]. The interpretation of results from correlation research may generate assumptions of causality. If disease is a predictor of health, and health has an effect on QoL, then therapies reducing the burden of disease are expected to improve QoL. On the other hand, if the patient's appraisal of QoL is not only an outcome, but also affects the perception of health, then the expectation that surgery can improve QoL may be too simplistic. In other words, reciprocal causality will interfere with the interpretation of heart surgery's effect on overall quality of life. Valid and reliable measures of QoL remain at risk of being labeled 'unresponsive' unless this latent controversy is understood and resolved. In clinical practice, evidence of reciprocal causality can support preoperative screening for patients who rate their QoL as poor, to guide complementary interventions during rehabilitation to ensure that outcomes following surgery are maximized. In this study, our aim was to investigate the existence of a reciprocal relationship between patients' assessment of quality of life and their appraisal of health.

### Theoretical framework

**Figure 1:**
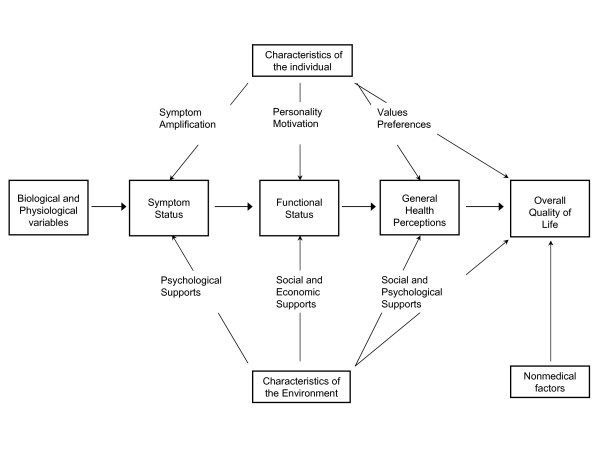
**A causal pathway model of health-related quality of life**. The horizontal arrows indicate the main, but not exclusive, direction of causality. Reproduced with permission from Wilson IB, Cleary PD. JAMA 1995;273:59–65. Copyright ^©^1995 American Medical Association.

In 1995, Wilson and Cleary proposed a causal pathway model to link clinical variables to QoL (Figure 1), in order to connect the field of objective measurement to that of subjective experience [5].

This model has influenced the analysis of data from cardiac [6-8] and other patient populations [9,10]. Wilson and Cleary structured outcomes along a continuum of increasing complexity from biological parameters through symptom status, functional status, general health perceptions and overall QoL [5]. General health perceptions reflect the functional status and symptoms such as angina pectoris [5], and are important for their predictive ability on the use of health care services as well as mortality [11]. Wilson and Cleary used the concepts health status and health-related QoL interchangeably in the description of their model. However, both concepts appear clearly separate from overall QoL, which represents "a stable synthesis of a wide range of experiences and feelings that people have" [5]. Interaction effects of individual and environmental characteristics may occur at each level of outcomes.

Previous research citing Wilson and Cleary has modeled unidirectional causal effects from general health perceptions towards overall QoL [6-10], under the assumption that the dominant path of causality is sufficient to guide data analysis. However, interpretation of results is conditional upon the absence of significant reciprocal effects. It is debatable whether QoL represents a summary outcome of different and situational life aspects, or a "top-down" individual disposition towards the evaluation of life aspects [12,13]. Integration of these theoretical positions in a reciprocal causality model of "top-down" personality factors and "bottom-up" situational variables has been proposed [14,15]. With repeated measurements of health status and overall QoL in patients undergoing heart surgery, an opportunity exists to challenge the conventional direction of causality illustrated in Figure 1, and assess the strength of causal relationships over time. If reciprocal causality is possible and the mechanisms can be explained, the Wilson and Cleary model must be accepted as more complex than previously recognized in correlation research.

## Methods

### Patient sample

The data set came from a previously reported [16] randomized clinical study of on-pump versus off-pump coronary artery bypass surgery. The parent study recruited and included 120 patients between 40 and 80 years of age, with stable angina pectoris and moderate or good left ventricular function. Exclusion criteria were ejection fraction < 30 % and/or renal failure (serum creatinine > 200 mmol/L), as well as patients unable to read, write or communicate verbally in Norwegian. The study protocol was approved by the Regional Ethics Committee, and patients provided written and informed consent. Five patients were lost to follow-up due to mortality (2 patients) and withdrawal (3 patients).

For the present path analysis, the patients constituted one group, as no significant effect of randomization to either treatment arm was found [16]. Complete sets of data were required for the analysis, resulting in the exclusion from analysis of seven more patients where one or more data points were missing. Thus, the patient sample for the present study included 108 complete sets out of 120 potential sets of patient data, representing individuals (81 % men) between 47 and 79 years (mean age 64.2 years). The patients were comparable to the parent study population on all subscales of the SF-36 health status survey. These individuals reported a median angina score of Canadian Cardiovascular Society class II [17], and 44 % had previously experienced myocardial infarction.

### Procedure

A study database provided demographic data and clinical parameters. The patients completed a questionnaire 4 times; at hospital admission before surgery (baseline), and 3, 6 (questionnaire sent by mail) and 12 months after surgery (follow-up visits at months 3 and 12). While the questionnaire also included outcome measures such as symptoms and functional status, the data analyzed in the present paper only concern overall QoL and general health perceptions. All in-patient assessments were scheduled before any further clinical or research diagnostics.

### Self-reported variables

#### Overall quality of life

Overall QoL can be measured in different ways depending on the substantive focus of investigation, such as happiness, well-being, life satisfaction [18]. The theoretical rationale and explicit ambition of the Wilson and Cleary model, integration of the biomedical and social sciences, suggested the use of life satisfaction instruments to represent overall quality of life [5]. Two instruments, Global Quality of Life (gQoL) and a Norwegian version of the Quality of Life Survey (QoLS-N), were used in order to assess the influence of methods' effects.

Global Quality of Life, previously used in epidemiological research [19], is a single-item overall appraisal of satisfaction with current life, scored on a seven step Likert-type scale: "Thinking about your life at the moment, would you say that you by and large are satisfied with life, or are you mostly dissatisfied?". The labeled response options ranged from 'very dissatisfied' to 'very satisfied'.

The QoLS-N is a 16-item scale reported as a single sum of item scores ranging from 16–112 points; higher scores indicating better QoL. It explores factors such as physical and material well-being, relationships with other people, social and civic activities, personal development, recreation, and independence [20]. Each item is scored on a seven-step scale with labeled response options that range from 'very dissatisfied' to 'very satisfied' in the validated translation [18]. Internal consistency (Cronbach's alpha) of the QoLS-N is reported at 0.86, with a test-retest reliability of 0.83 [21]. In this study, internal consistency was 0.83 at baseline and 0.90 at all subsequent time-points.

#### General health perceptions

We used the General Health subscale from the Short Form 36 (SF-36 version 1.2) as a single indicator. The 5 general health items cover current health, health outlook, and resistance to illness. Scores range from 0–100 points; higher scores indicate better health. Internal consistency (Cronbach s alpha) for this subscale has been reported at 0.84 [22] and varied in our study from 0.73 at baseline and three months' assessment, to 0.78 (six months) and 0.81 (twelve months). General health perceptions are associated with physical, mental and social health domains [23].

### Statistical analysis

**Figure 2:**
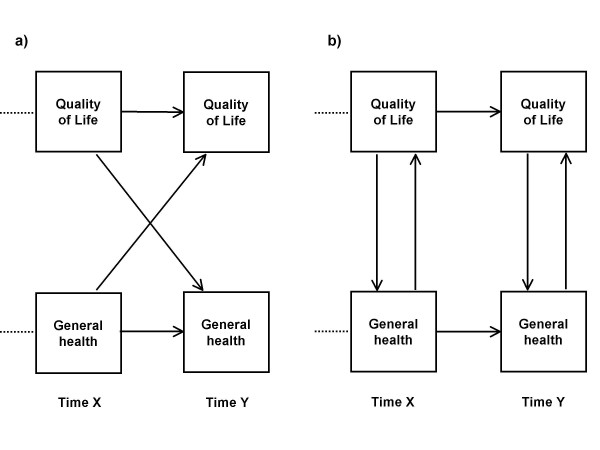
**Reciprocal causal paths, illustrating a) cross-lagged effects and b) simultaneous effects**. Single arrows indicate causal paths. Only two time-points are illustrated, while four time-points were analyzed in all models reported in this paper.

We modeled causal paths with longitudinal data between single indicator variables for overall quality of life and general health perceptions. The path analysis used structural equation modeling [24] where all 4 time-points were represented in all models tested. This method allows the inclusion of feedback or reciprocal paths in addition to unidirectional causal effects [25] and is therefore more appropriate for our study than standard multiple regression technique. Figure 2 illustrates the two different sets of reciprocal relationships that were modeled. Cross-lagged components model the causal effect as observed at a later point in time (Figure 2a), while simultaneous components are observed at the same time (Figure 2b).

Structural equation modeling does not prove causality, but it tests whether the data set, with its inherent covariance structure, supports or rejects the postulated effects. Thus, the data matrix under analysis is the set of covariances between all pairs of variables. The interrelationships of the observed variables are specified in structural equations by the researcher, according to hypotheses and theories. Adding, removing or changing the direction of an effect (arrow in Figure 2) means changing the set of regression equations. The combination of all equations form a model, and the fit or appropriateness of this model is tested by analyzing all equations simultaneously, looking at the whole landscape rather than the individual parts. The result of analysis is expressed as a set of fit indices, indicating how well the specified model fits the observed reality. The task of interpretation is to accept, reject, or possibly modify the paths included in the model.

Scoring of the SF-36 was completed according to the manual [23] using the SPSS version 12.0 (SPSS Inc., Chicago IL). To analyze the extent of selective attrition, χ^2 ^and t-tests for independent samples were used. Histograms and descriptive statistics for the individual variables were screened for deviations from normality. The distribution of scores indicated reasonable compliance with the assumptions of linear modeling. The covariance matrix was analyzed using maximum likelihood estimation in Lisrel version 8.72 (Scientific Software International, Lincolnwood IL). We allowed for each effect component to vary over time. First, we modeled the time-lagged effects between general health perceptions and overall QoL over 4 time-points. We included correlations between variables measured at the same occasion to take into account the presence of confounding variables [25,26]. The second model included the cross-lagged effects between the different variables to the following time-point of assessment. Finally, a third model was introduced to evaluate simultaneous effects, where the correlation between variables measured within one time-point in the previous models was replaced by reciprocal causal effects. A series of model fit characteristics were used to evaluate the adequacy of different causal models: (i) χ^2 ^analysis, testing that the model is not significantly different (p > 0.05) from the underlying population covariance matrix, (ii) comparative fit index (CFI) above 0.90, (iii) root mean square error approximation (RMSEA) indicating acceptable (RMSEA < 0.08) or good (< 0.05) fit of the residuals, and (iv) standardized root mean square residuals (SRMR) less than 0.10 [24]. The p of Close Fit tested the null hypothesis of RMSEA < 0.05; a non-significant test implies acceptable model fit. The significance of the cross-lagged effects and the equivalence of cross-lagged or simultaneous effects were evaluated with a χ^2 ^difference test using a critical value (alpha) of 5 %.

## Results

General health and QoL measurements at all time-points are presented in Table 1, and their bivariate relationships in Table 2. Relative to preoperative status, the mean improvement in general health score at all three follow-up points ranged from 9.5 (SD 18.8) to 12 (SD 18.6) points. At the group level, the greatest improvement in general health occurred from baseline to 3 months after surgery, although at the individual level, one third (32.4 %) of the individual patients reported no change or decline.

At all assessments, the individual patient scores on the global Quality of Life item (gQoL) covered the full scale range from 1 to 7. The group mean gQoL improved from 5.0 before surgery to 5.6 at 12 months' follow-up (Table 1). Overall QoL scores measured with the QoLS-N also demonstrated individual variation; the group mean at baseline was 86.5 points increasing to 88.1 after 12 months, while individual scores ranged from 52.6 to 112 points during the same time-span.

**Table 1:** General health and overall quality of life at 4 time-points (n = 108)

	Baseline		3 months		6 months		12 months	
				
General health	57.7	(21.1)	69.7	(18.8)	67.2	(19.7)	68.7	(21.3)
gQoL	5.0	(1.1)	5.6	(0.9)	5.5	(0.9)	5.6	(0.9)
QoLS-N	86.5	(10.2)	88.0	(10.3)	87.4	(11.0)	88.1	(10.3)

Each observation is presented as mean (standard deviation).

For all three scales, higher scores indicate better health or quality of life.

General health scale range 0–100.

gQoL scale range 1–7.

QoLS-N scale range 16–112.

**Table 2:** Bivariate relationships of general health perceptions and overall quality of life at four time-points

	Baseline			3 months			6 months			12 months		
	General health	gQoL	QoLS-N	General health	gQoL	QoLS-N	General health	gQoL	QoLS-N	General health	gQoL	QoLS-N
				
Baseline												
General health	*446*	.32*	.38*	.57*	.27*	.18	.58*	.24	.20	.54*	.22	.24
gQoL		*1.25*	.42*	.37*	.32*	.23	.36*	.43*	.34*	.35*	.46*	.35*
QoLS-N			*104*	.28*	.22	.58*	.35*	.30*	.59*	.21	.24	.59*
3 months												
General health				*354*	.55*	.42*	.71*	.39*	.30*	.75*	.46*	.42*
gQoL					*.76*	.52*	.44*	.53*	.30*	.47*	.54*	.46*
QoLS-N						*107*	.36*	.37*	.61*	.35*	.37*	.66*
6 months												
General health							*387*	.53*	.54*	.66*	.41*	.51*
gQoL								*.81*	.57*	.37*	.62*	.42*
QoLS-N									*120*	.24	.43*	.64*
12 months												
General health										*453*	.43*	.49*
gQoL											*.80*	.52*
QoLS-N												*106*

The diagonal (italic) represents variance; the upper diagonal contains Pearson correlation coefficients, asterisks indicate p = 0.01. Common to all three scales, higher scores indicate better health or quality of life.

### Structural equation modeling of global Quality of Life (gQoL)

**Figure 3:**
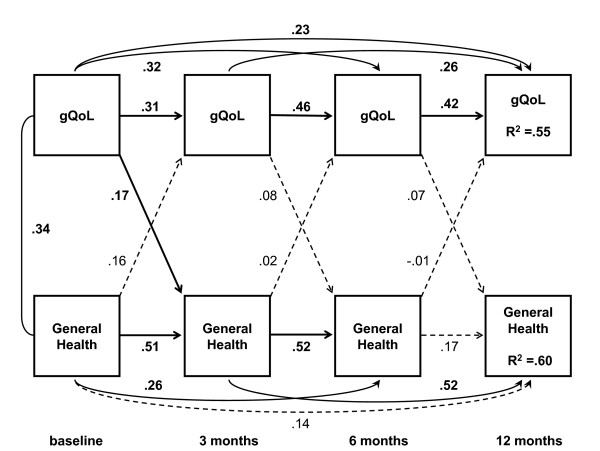
**Cross-lagged model**. This model demonstrates bidirectional causal paths from one time-point to the following, from general health towards overall quality of life as well as from overall quality of life towards general health perceptions. One of these cross-lagged paths is statistically significant; the path from baseline gQOL towards 3 months' scores on the SF-36 General health subscale. Together with the cross-lagged paths, the model also includes strong serial associations over time within each variable except between 6 and 12 months general health perceptions. Straight and curved single arrows indicate the causal paths modeled. The corresponding decimals are standardized regression coefficients. Bold face coefficients indicate p < 0.05 while broken lines are used for paths with a corresponding p ≥ 0.05. The curved line between baseline variables represents a correlation; the number is the corresponding correlation coefficient. Model fit indices are summarized in Table 3. Figures 3 and 4 are available in more detailed versions as Additional files 1 (Additional file 1_xlagged_gQOL.pdf) and 2 (Additional file 2_simultaneous_gQOL.pdf) with this paper, including the unstandardized parameter estimates.

**Figure 4:**
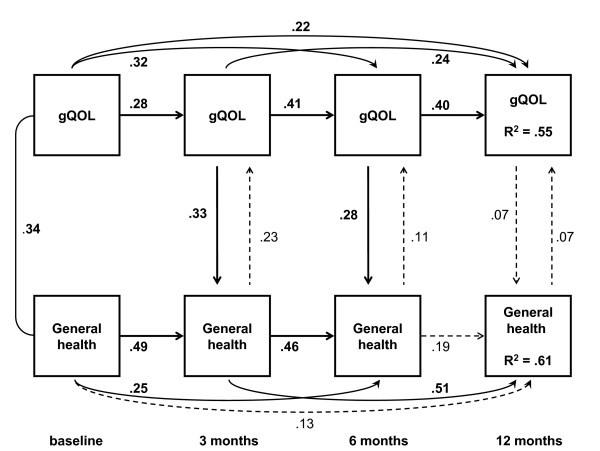
**Simultaneous effects model**. This model demonstrates bidirectional causal paths at each time-point observed after baseline. The path from general health towards overall quality of life occurs at the same time as the path from overall quality of life towards general health perceptions is observed. Two of the six simultaneous paths are statistically significant in the direction from 3 and 6 months' gQOL towards the corresponding scores on the SF-36 General health subscale. As with the cross-lagged paths, this model includes strong serial associations over time within each variable except between 6 and 12 months' general health perceptions. Straight and curved single arrows indicate the causal paths modeled. The corresponding decimals are standardized regression coefficients. Bold face coefficients indicate p < 0.05 while broken lines are used for paths with a corresponding p ≥ 0.05. The curved line between baseline variables represents a correlation; the number is the corresponding correlation coefficient. Model fit indices are summarized in Table 3. For correct model specification of simultaneous effects, the present baseline assessment provides exogenous and correlated variables, without simultaneous effects at this time-point.

Full versions of the cross-lagged and simultaneous path models, with unstandardized estimates of the paths as well as correlations of the error variances of the variables, are available under Additional files. The abbreviated versions in Figure 3 and 4 serve to outline the main results with standardized regression coefficients.

We started by estimating a model with longitudinal paths connecting all assessments within each of the two outcome measures at all time-points, but no causal paths between general health and overall QoL. The analysis provided acceptable model fit and consequently, this model served as a reference model from which improvements through the addition of hypothesized causal paths could be evaluated (Table 3). We also tested an alternative and more parsimonious lagged effects model, where the only paths specified were the connections from one time-point to the immediate next within each variable, omitting the bridging connections between baseline and 6 months, baseline and 12 months, and 3 months and 12 months' assessments. This model fitted the data poorly and was discarded.

The cross-lagged model added reciprocal effects from the previous to the next assessment between QoL and general health perceptions, and the standardized regression coefficients of these causal paths are presented in Figure 3 (see also Additional file 1: Additional file 1_xlagged_gQOL.pdf). A statistically significant cross-lagged effect from overall QoL at baseline to the three months assessment of general health was present, indicating independent predictor properties of the baseline QoL appraisal during the greatest change in general health status. This model fitted the data well (Table 3) although the change in χ^2 ^compared to the lagged model was not statistically significant. The simultaneous reciprocal model (Figure 4) demonstrated best fit and, by chi-square test, a significant model improvement, with significant path coefficients observed at three and six months after surgery from overall QoL toward general health perceptions (see also Additional file 2: Additional file 2_simultaneous_gQOL.pdf). To contrast this analysis with an assumption of no causal effects, we set the bidirectional paths within each time-point to equal size. This resulted in an unidentifiable model.

**Table 3:** Model fit indices

	χ^2^	df	p	RMSEA	CFI	SRMR	Δχ^2^	Δdf
gQoL								
1. Lagged effects only	18.55	12	.100	.072 ^a^	.990	.125	reference	
2. Cross-lagged effects	7.71	6	.260	.052 ^a^	.997	.023	10.84	6
(Figure 3)								
3. Simultaneous effects	9.76	9	.371	.028 ^a^	.998	.026	8.79 ^b^	3
(Figure 4)								

QoLS-N								
4. Lagged effects only	17.50	12	.132	.066 ^a^	.990	.076	reference	
5. Cross-lagged effects	7.21	6	.302	.044 ^a^	.998	.022	10.29	6
6. Simultaneous effects	17.49	9	.042	.095 ^a, c^	.986	.066	.01	3

^a ^p of Close Fit >.05, the null hypothesis is RMSEA <.05.

^b ^*P *< .05

^c ^The 90% confidence interval is .018 to .161

CFI Comparative Fit Index, values > 0.90 indicates a good fit of the model.

RMSEA Root Mean Square Error of Approximation, values < 0.08 indicating acceptable, and < 0.05 good fit of the residuals.

SRMR Standardized Root Mean Residual, values < 0.10 indicating good fit.

Δdf Difference in degrees of freedom from reference model

Δχ^2 ^Difference in chi-square from reference model chi square

### Structural equation modeling of the Quality of Life Survey (QoLS-N)

**Figure 5:**
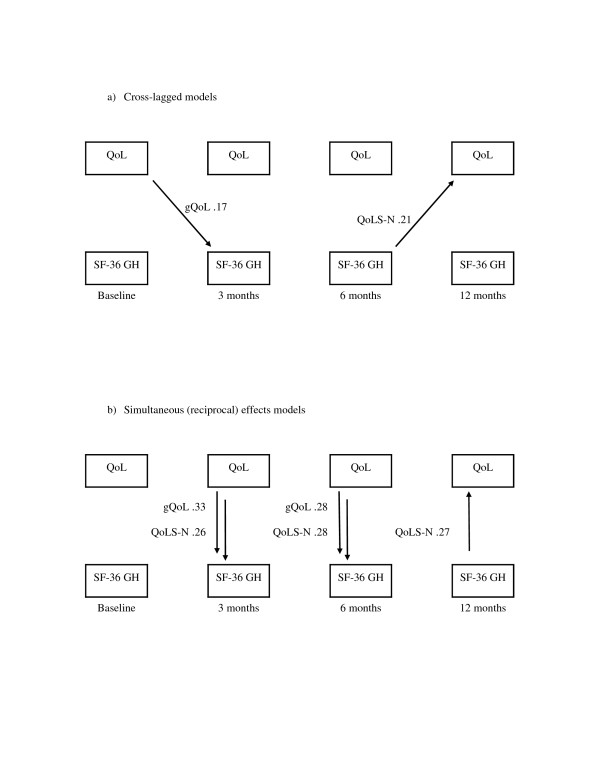
**Comparison of significant a) cross-lagged and b) simultaneous paths from two sets of modeling with two different quality of life instruments: gQoL and QoLS-N**. Figure 5 summarizes only the statistically significant paths observed between General Health and overall Quality of Life, indicated as arrows in the direction of causality. The causal paths are labeled with their corresponding QoL instrument and standardized regression coefficients derived from structural equations. Paths within each concept from one time-point to another, for example from General Health at three months to General Health at 12 months, are not drawn. See Table 3 for model fit indices, and Additional files 1 through 4 for separate model parameters.

We re-ran the previous analyses with the QoLS-N results (Table 3). Full versions of the cross-lagged and simultaneous path models are available as Additional files 3 and 4 (Additional file 3_xlagged_QOLSN.pdf, and Additional file 4_simultaneous_QOLSN.pdf). By fitting identical models with the two instruments, it was possible to assess the extent of instrument-specific results, which constitutes a step towards cross-validation (Figure 5).

In the cross-lagged model, the path from general health at six months to QoL at 12 months was statistically significant. Each point increase in general health at six months, resulting from all directional paths assigned, was associated with 0.11 point increase in QoLS-N at one year after surgery. In the simultaneous reciprocal model, significant standardized regression coefficients from QoL to general health dominated at 3 and 6 months after surgery (0.26 and 0.28, respectively), while the reverse effect from general health towards QoL (0.27) was present at one year after surgery. However, the model fit indices suggested that the cross-lagged model was superior to the model allowing simultaneous and bidirectional causal paths (Table 3). Cross-lagged effects also demonstrated better model fit than two unidirectional and simultaneous effects models, either according to the conventional paths of the Wilson and Cleary model or with reversed causation only – from QoL towards general health. An extended version of Table 3 details the latter results and is available on request.

## Discussion

In this study, structural equation modeling supported the existence of reciprocal causal paths between general health perceptions and overall QoL. Our longitudinal analysis indicated that changes in general health perceptions may be conditional upon as well as contributing to the appraisal of overall QoL. The clinical implication is that the evidence used for preoperative counseling on expected changes in symptoms and functioning, should not be extrapolated to health in general or life satisfaction following coronary artery bypass surgery. Restraining the level of abstraction to outcomes that are conceptually closer to clinical parameters and the surgical intervention, such as symptoms and functional status domains [5], may prevent misunderstandings and facilitate joint decision-making between patient and provider. Bearing on research, the present study describes a potential source of error when interpreting cross-sectional associations between overall QoL, general health, and heart surgery.

We based our analysis on research demonstrating that general health perceptions and overall QoL represent conceptually and empirically distinct dimensions [27]. Previous studies of heart patients where the Wilson and Cleary model has been evaluated as a conceptual framework, present different interpretations of overall QoL: either as life satisfaction similar to the present study [6], or by referring to health-related QoL (HQoL) [28], or disease-specific health-related QoL [7]. If the main causal direction from general health perceptions towards overall QoL were dominant, blurring of the conceptual distinction between health status or HQoL and overall QoL would be less consequential, although possibly not desirable [3]. However, our results indicate a more complex causal network, which precludes conceptualizing overall QoL as health or subsumed within health. Examples from neighbor research fields expand on the causal networks involved, where explanatory variables associated with overall QoL include a genetic component [29,30], personality trait characteristics [31], and – in cardiac patients – life orientation or Sense of Coherence [32].

In our study, the regression coefficients between general health perceptions and overall QoL did not demonstrate a stable pattern. This variation may represent true variation of structurally stable constructs, or there may be unidentified structural variation due to response shifts from changing values or beliefs of respondents [33], possibly mediated by alterations in cognitive processing after heart surgery. Sample size was in this study insufficient for modeling of latent variables to control for time invariance of item factor loadings on each construct. However, we used instruments where the variance was fully modeled – as in the case of the single-item gQoL – or where the factor structure and reliability has been extensively reported across patient groups. The adequacy of the lagged effects models suggested that longitudinal construct validity was satisfactory. A homeostatic concept of subjective well-being offers a possible explanation to the changing "bottom-up" and "top-down" causal paths over situations and time. It has been suggested that mechanisms to restore homeostasis are triggered when a challenge to an individual set point occurs from contributing domains [13]. Severe health impairments may trigger through negative feedback, whereas positive health transitions following surgery may alter the relative strength of bidirectional paths connecting health perceptions and QoL. In the present study, the patients were in a transition from a preoperative state through rehabilitation. During the period of greatest change in general health perceptions, from preoperative status to three months after surgery, paths from overall QoL towards general health appeared dominant in the simultaneous effects models (gQoL and QoLS-N) as well as in the cross-lagged model using gQoL observations. In contrast, the cross-lagged QoLS-N model was indifferent from baseline to three months after surgery. Furthermore, from six months to one year after surgery and during less magnitude of health transition, the regression coefficients in the conventional direction from general health perceptions towards overall QoL were significant in the cross-lagged and simultaneous effects QOLS-N models (Figure 5, Additional file 3: Additional file 3_xlagged_QOLSN, and Additional file 4: Additional file 4_simultaneous_QOLSN).

Variation between regression coefficients were in the present study associated with the choice of overall QoL instrument. While a consensus exists as to how QoL should be assessed, i.e. as a subjective appraisal obtained by asking the patient [34], there is no gold standard or reference criterion for evaluation of content validity of overall QoL instruments [35]. In our observations, we selected measures that emphasize life satisfaction as a critical component of overall QoL. It should be noted that Wilson and Cleary [5] also cite subjective well-being and happiness along with life satisfaction as representative for overall QoL, although their paper does not enter the discussion on structural relationships of indicators of overall QoL. The correlation matrix of Table 2 indicates only a moderate overlap of content between the gQoL and the QoLS-N. Their intercorrelation coefficients remain below 0.60, and the extent of common methods variance is unknown. Two complementary explanations may be offered for the modest strength of association and the different results obtained when modeling with different instruments: First, compared to the QoLS-N, the single-item gQoL emphasizes "top-down" effects towards general health perceptions during changing health conditions. It is possible that the single question favors a life orientation response, as the response options neither are anchored to specific life domains nor impose any assumptions of weighting due to the number or order of items. Conversely, the sum score of the QoLS-N may represent a bottom-up perspective of overall QoL as a sum of experiences and appraisals. However, although the selection of items is empirically grounded [20], each item is given equal weight in the summary score and this may not adequately reflect the preferences and priorities held by respondents.

Second, one may question whether the gQoL and the QOLS-N represent the same latent variable, life satisfaction. Exploring their relationships, we correlated the gQoL at baseline and at one year after surgery with a three-factor solution of the QOLS derived from analysis of healthy subjects' responses [20]. Amongst these factors, Health and Functioning demonstrated the greatest strength of association to the gQoL, followed by Relationships and Material Well-Being and finally Personal, Social and Community Commitment. Of note, the QoLS-N scale contains one item specifying physical health. To control for untoward loss of variance and inflated regression coefficients between the observed variables in our analyses, we ran separate models with a 15-item modification of the QoLS-N score in which the health item was deleted. No substantial change in model fit was observed (data available on request).

Some limitations of this study should be acknowledged. We assumed that the baseline values of our observed variables carried adequate adjustment for numerous candidate background variables such as gender, age, socioeconomic status, level of education and co-morbidity. A larger sample size would allow for more parameters and variables – observed or latent – to be included. However, our analysis used the p of Close Fit indicator (see legend, Table 3) to provide an estimate of sufficient power to detect poor model fit due to misspecification. As this study is an early investigation of reciprocal effects, we could not locate publications that could validate the timing of assessments as more or less sensitive to the causal paths investigated.

## Conclusion

Unidirectional models of causality are inadequate to explain the effect of cardiac surgery on overall QoL. Overall quality of life can causally influence as well as be an outcome of health status after coronary artery bypass surgery. Our analysis substantiates the potential for reciprocal effects within the Wilson and Cleary model. This study offers a pilot design for confirmatory modeling with more frequent sampling of a larger patient population.

## Abbreviations

gQoL Single-item Global Quality of Life question

QoL Quality of Life

QoLS-N Quality of Life Survey-Norwegian

SF-36 GH Short Form 36 General Health subscale

## Competing interests

The author(s) declare that they have no competing interests.

## Authors' contributions

LM initiated this paper as part of a larger study of patient reported outcomes and drafted the manuscript together with MV, who provided statistical advice. MA participated in data collection. All authors critiqued revisions of the paper and approved the final manuscript. EF, BRH and AKW supervised LM and MA; EF was principal investigator for the research program on off-pump versus on-pump coronary artery bypass surgery.

## Supplementary Material

Additional file 1 Cross-lagged model with Global Quality of Life (gQOL) displaying unstandardized and standardized estimates, together with correlation coefficients between error variances. Click here for file

Additional file 2 Simultaneous reciprocal effects model with Global Quality of Life (gQOL) displaying unstandardized and standardized estimates. Click here for file

Additional file 3 Cross-lagged model with Quality of Life Scale – Norwegian version (QOLS-N) displaying unstandardized and standardized estimates, together with correlation coefficients between error variances. Click here for file

Additional file 4 Simultaneous reciprocal effects model with Quality of Life Scale – Norwegian version (QOLS-N) displaying unstandardized and standardized estimates. Click here for file

## References

[B1] Eagle KA, Guyton RA, Davidoff R, Edwards FH, Ewy GA, Gardner TJ, Hart JC, Herrmann HC, Hillis LD, Hutter AM, Lytle BW, Marlow RA, Nugent WC, Orszulak TA (2004). ACC/AHA 2004 guideline update for coronary artery bypass graft surgery: a report of the American College of Cardiology/American Heart Association Task Force on Practice Guidelines (Committee to Update the 1999 Guidelines for Coronary Artery Bypass Graft Surgery). Circulation.

[B2] Gibbons RJ, Abrams J, Chatterjee K, Daley J, Deedwania PC, Douglas JS, Ferguson TB, Fihn SD, Fraker TD, Gardin JM, O'Rourke RA, Pasternak RC, Williams SV, Gibbons RJ, Alpert JS, Antman EM, Hiratzka LF, Fuster V, Faxon DP, Gregoratos G, Jacobs AK, Smith SC ACC/AHA 2002 Guideline update for the management of patients with chronic stable angina: a report of the American College of Cardiology/American Heart Association Task Force on Practice Guidelines (Committee to Update the 1999 Guidelines for the Management of Patients with Chronic Stable Angina). http://www.acc.org/qualityandscience/clinical/guidelines/stable/stable_clean.pdf.

[B3] Tennant A (1995). Quality of life–a measure too far?. Ann Rheum Dis.

[B4] Holmes S (2005). Assessing the quality of life-reality or impossible dream? A discussion paper. Int J Nurs Stud.

[B5] Wilson IB, Cleary PD (1995). Linking clinical variables with health-related quality of life. A conceptual model of patient outcomes. JAMA.

[B6] Janz NK, Janevic MR, Dodge JA, Fingerlin TE, Schork MA, Mosca LJ, Clark NM (2001). Factors influencing quality of life in older women with heart disease. Med Care.

[B7] Hofer S, Benzer W, Alber H, Ruttmann E, Kopp M, Schussler G, Doering S (2005). Determinants of health-related quality of life in coronary artery disease patients: a prospective study generating a structural equation model. Psychosomatics.

[B8] Arnold R, Ranchor AV, Sanderman R, Kempen GIJM, Ormel J, Suurmeijer TPBM (2004). The relative contribution of domains of quality of life to overall quality of life for different chronic diseases. Qual Life Res.

[B9] Wettergren L, Bjorkholm M, Axdorph U, Langius-Eklof A (2004). Determinants of health-related quality of life in long-term survivors of Hodgkin's lymphoma. Qual Life Res.

[B10] Sousa KH, Holzemer WL, Henry SB, Slaughter R (1999). Dimensions of health-related quality of life in persons living with HIV disease. J Adv Nurs.

[B11] Kelli L, Heller DA, Ahern FM, Gold CH (2002). Relationship of health-related quality of life to health care utilization and mortality among older adults. Aging Clin Exp Res.

[B12] Diener E (1984). Subjective well-being. Psychol Bull.

[B13] Cummins RA (2003). Normative Life Satisfaction: Measurement Issues and a Homeostatic Model. Soc Ind Res.

[B14] Brief AP, Butcher AH, George JM, Link KE (1993). Integrating bottom-up and top-down theories of subjective well-being: The case of health. J Pers Soc Psychol.

[B15] Lance CE, Mallard AG, Michalos AC (1995). Tests of the causal directions of global-life facet satisfaction relationships. Soc Ind Res.

[B16] Mathisen L, Hol PK, Lingaas PS, Lundblad R, Rein KA, Tonnessen TI, Mork BE, Svennevig JL, Wahl AK, Hanestad BR, Fosse E (2005). Patient reported outcome after randomization to on-pump versus off-pump coronary artery surgery. Ann Thorac Surg.

[B17] Campeau L (1976). Grading of angina pectoris. Circulation.

[B18] Wahl AK, Rustoen T, Hanestad BR, Lerdal A, Moum T (2004). Quality of life in the general Norwegian population, measured by the Quality of Life Scale (QOLS-N). Qual Life Res.

[B19] Holmen J, Midthjell K (1990). The North Trøndelag Survey 1984–86.

[B20] Burckhardt C, Anderson K (2003). The Quality of Life Scale (QOLS): Reliability, validity, and utilization. Health Qual Life Outcomes.

[B21] Wahl A, Burckhardt C, Wiklund I, Hanestad BR (1998). The Norwegian version of the Quality of Life Scale (QOLS-N). Scand J Caring Sci.

[B22] Loge JH, Kaasa S (1998). Short form 36 (SF-36) health survey: normative data from the general Norwegian population. Scand J Soc Med.

[B23] Ware JE, Snow KK, Kosinski M, Gandek B (1993). SF-36 Health Survey: Manual and interpretation guide.

[B24] Kline RB (2005). Principles and practice of structural equation modeling.

[B25] Finkel SE (1995). Causal analysis with panel data.

[B26] Zapf D, Dormann C, Frese M (1996). Longitudinal studies in organizational stress research: a review of the literature withreference to methodological issues. J Occup Health Psychol.

[B27] Smith KW, Avis NE, Assmann SF (1999). Distinguishing between quality of life and health status in quality of life research: a meta-analysis. Qual Life Res.

[B28] Heo S, Moser DK, Riegel B, Hall LA, Christman N (2005). Testing a published model of health-related quality of life in heart failure. J Card Fail.

[B29] Tellegen A, Lykken DT, Bouchard TJJ, Wilcox KJ, Segal NL, Rich S (1988). Personality similarity in twins reared apart and together. J Pers Soc Psychol.

[B30] Roysamb E, Tambs K, Reichborn-Kjennerud T, Neale MC, Harris JR (2003). Happiness and health: environmental and genetic contributions to the relationship between subjective well-being, perceived health, and somatic illness. J Pers Soc Psychol.

[B31] DeNeve KM, Cooper H (1998). The happy personality: A meta-analysis of 137 personality traits and subjective well-being. Psychol Bull.

[B32] Motzer SU, Stewart BJ (2005). Sense of coherence as a predictor of quality of life in persons with coronary heart disease surviving cardiac arrest. Res Nurs Health.

[B33] Rapkin BD, Schwartz CE (2004). Toward a theoretical model of quality-of-life appraisal: Implications of findings from studies of response shift. Health Qual Life Outcomes.

[B34] Fayers PM, Machin D (2000). Quality of life: Assessment, analysis and interpretation.

[B35] Anderson KL, Burckhardt CS (1999). Conceptualization and measurement of quality of life as an outcome variable for health care intervention and research. J Adv Nurs.

