# Supplementary figures and images for: Quality of life can both influence and be an outcome of general health perceptions after heart surgery
Source: Health Qual Life Outcomes. 2007 May 24;5:27. doi: 10.1186/1477-7525-5-27 (PMC1892007; doi:10.1186/1477-7525-5-27)

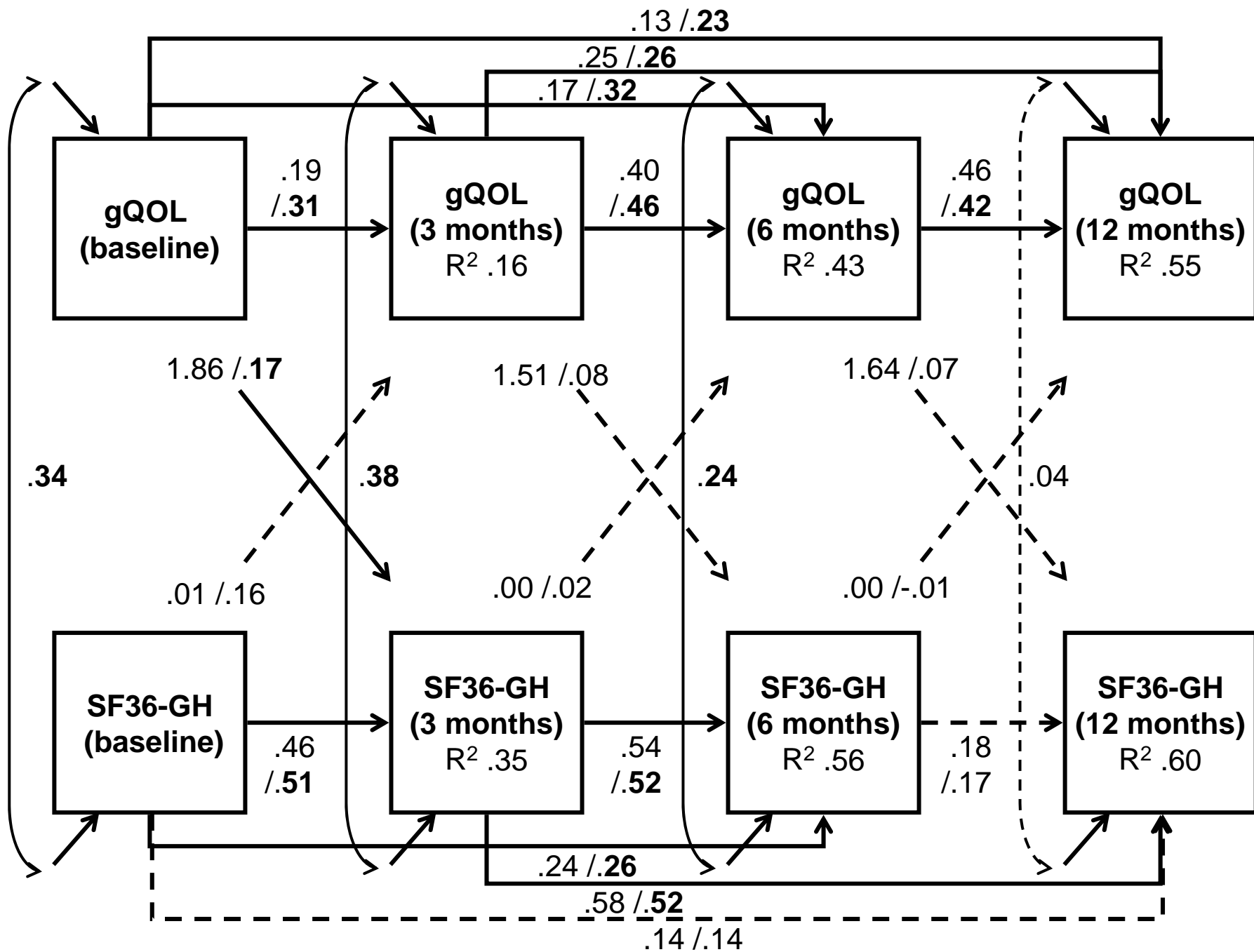

Supplement: Additional file 1 — Cross-lagged model with Global Quality of Life (gQOL) displaying unstandardized and standardized estimates, together with correlation coefficients between error variances. [file 1477-7525-5-27-S1.pdf]

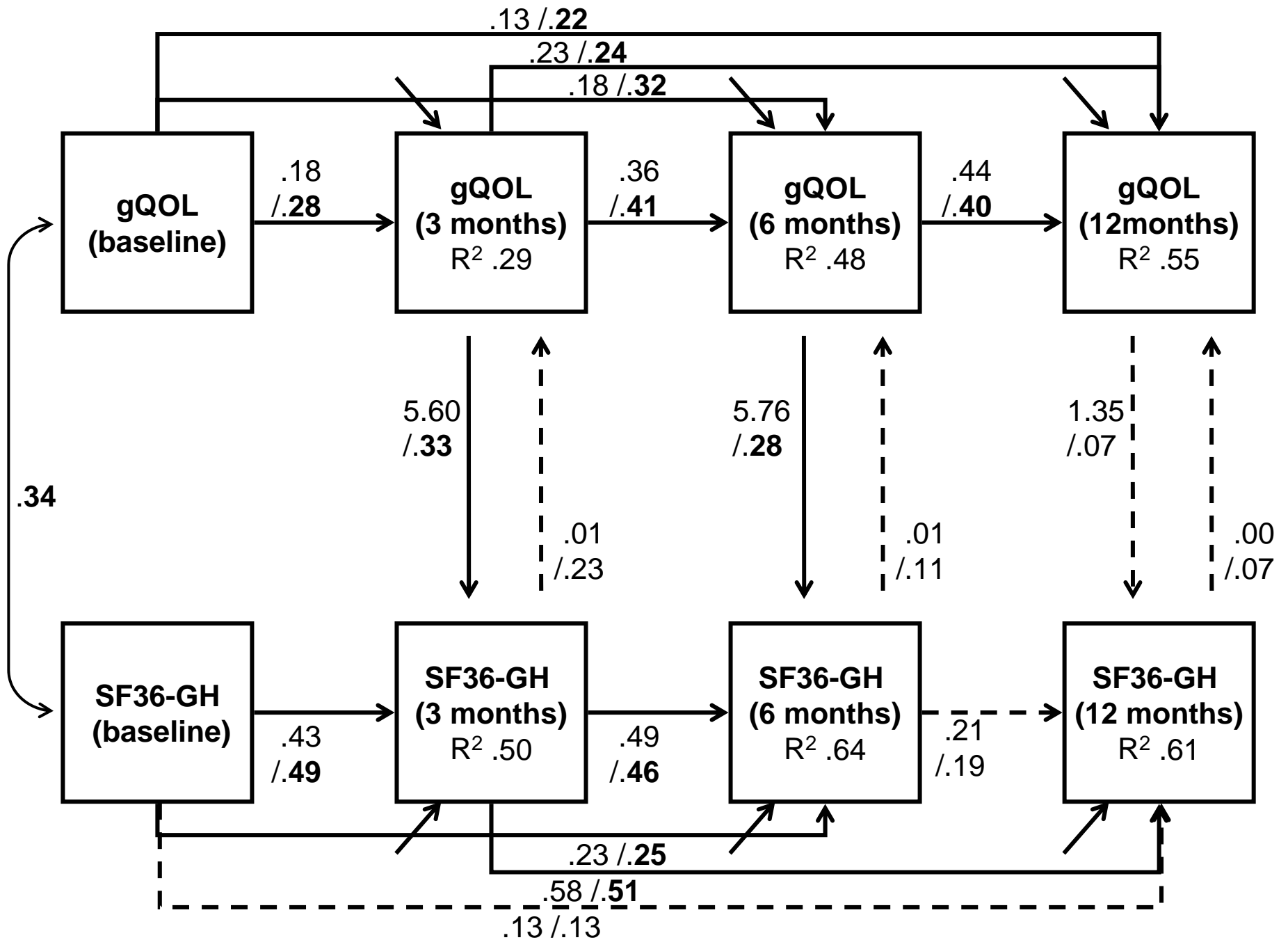

Supplement: Additional file 2 — Simultaneous reciprocal effects model with Global Quality of Life (gQOL) displaying unstandardized and standardized estimates. [file 1477-7525-5-27-S2.pdf]

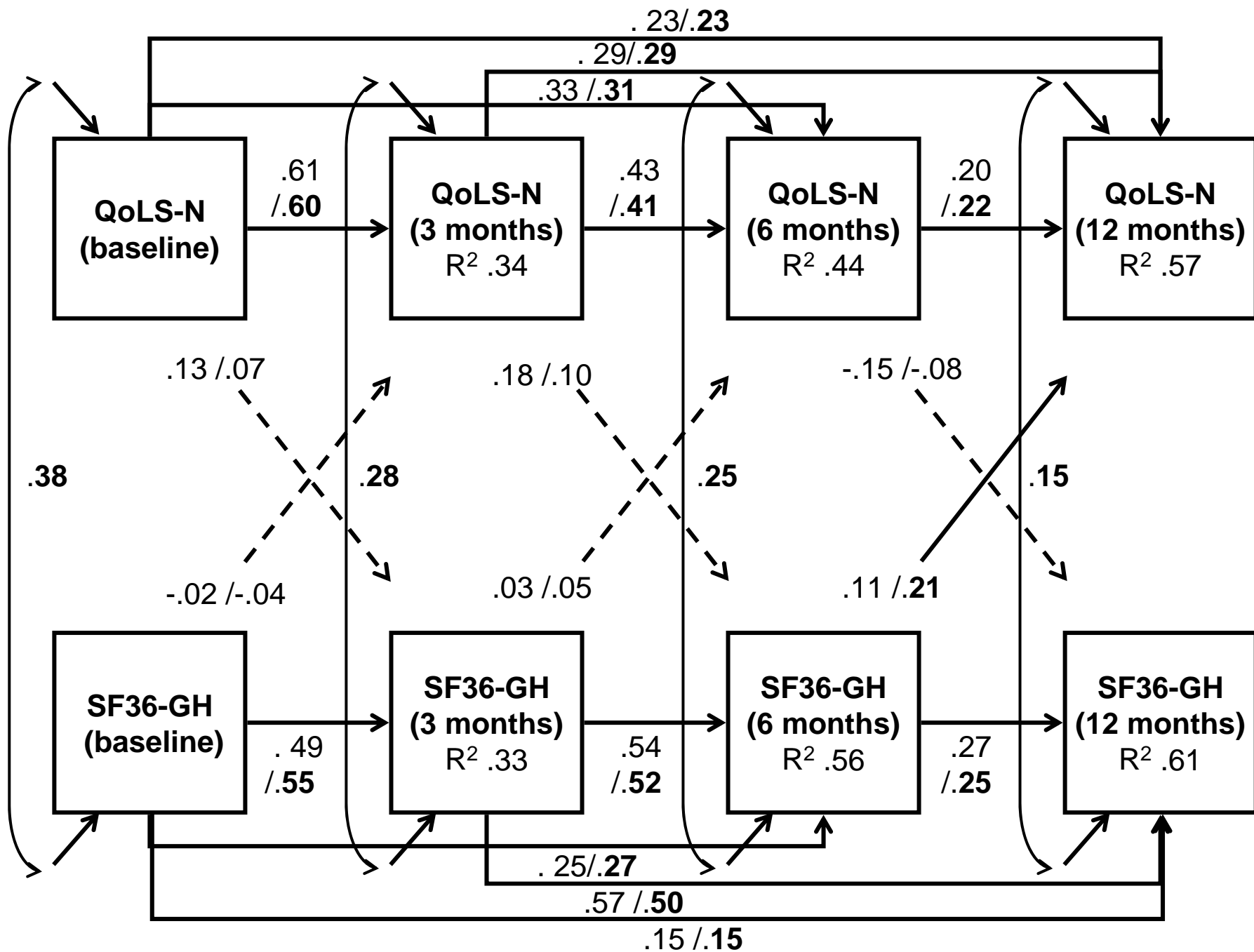

Supplement: Additional file 3 — Cross-lagged model with Quality of Life Scale – Norwegian version (QOLS-N) displaying unstandardized and standardized estimates, together with correlation coefficients between error variances. [file 1477-7525-5-27-S3.pdf]

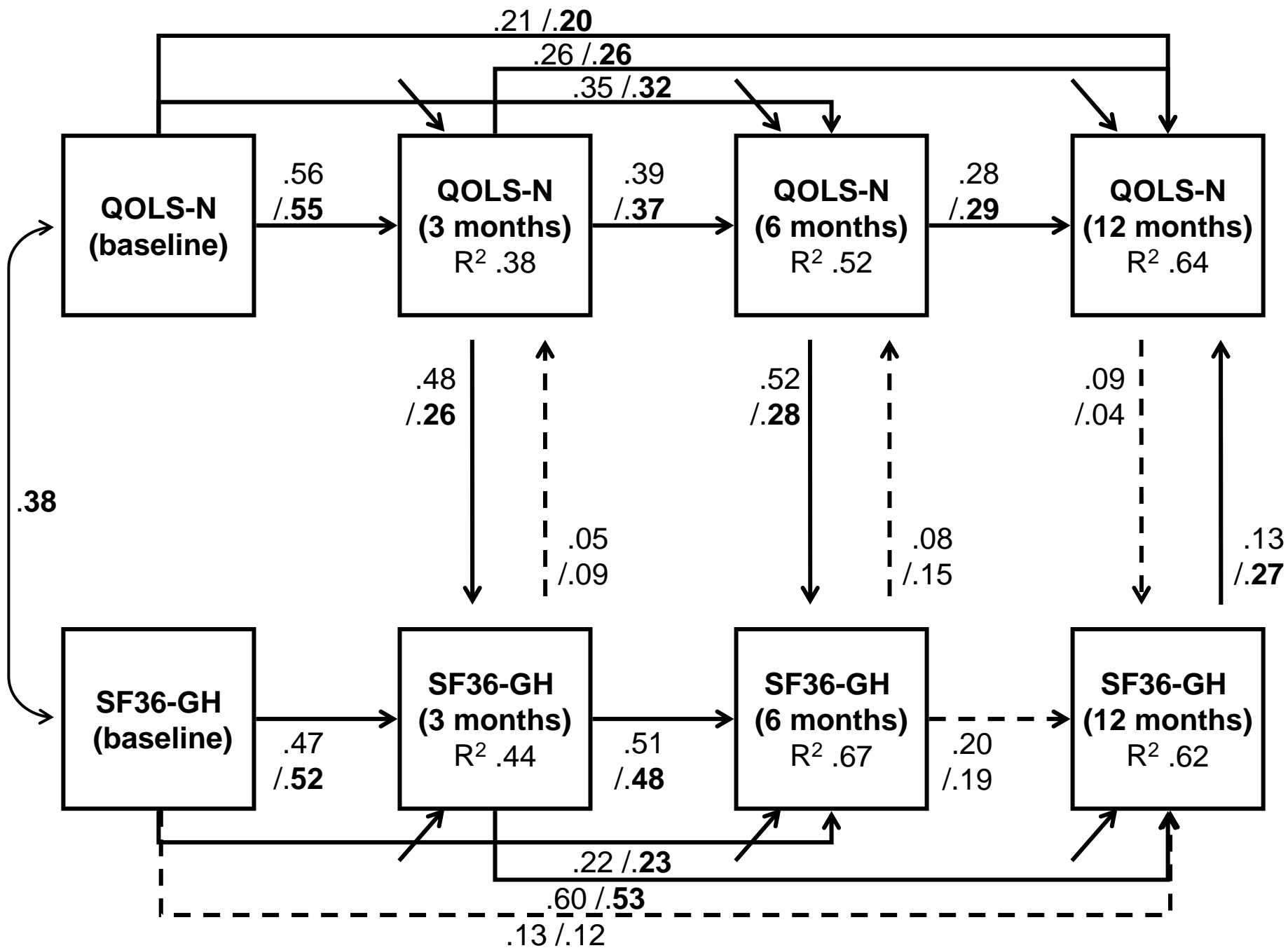

Supplement: Additional file 4 — Simultaneous reciprocal effects model with Quality of Life Scale – Norwegian version (QOLS-N) displaying unstandardized and standardized estimates. [file 1477-7525-5-27-S4.pdf]
